# Adaptations in Mitochondrial Function Induced by Exercise: A Therapeutic Route for Treatment-Resistant Depression

**DOI:** 10.3390/ijms26178697

**Published:** 2025-09-06

**Authors:** Arnulfo Ramos-Jiménez, Mariazel Rubio-Valles, Javier A. Ramos-Hernández, Everardo González-Rodríguez, Verónica Moreno-Brito

**Affiliations:** 1 Institute of Biomedical Sciences, Autonomous University of Ciudad Juarez, Ciudad Juarez Campus, Chihuahua 32310, Mexico; 2 Faculty of Physical Culture Sciences, Autonomous University of Chihuahua, University Circuit, Campus II, Chihuahua 31125, Mexico; p305510@uach.mx; 3 Faculty of Medicine, Autonomous University of Nuevo Leon, University Campus, Monterrey 64460, Mexico; javier.ramoshrn@uanl.edu.mx; 4 Faculty of Medicine, Autonomous University of Chihuahua, University Circuit, Campus II, Chihuahua 31125, Mexico; evegonzal@uach.mx (E.G.-R.); vmoreno@uach.mx (V.M.-B.)

**Keywords:** treatment-resistant depression, mood disorders, physical exercise, sedentarism, mitochondrial dynamics, neuroplasticity, neurophysiological disturbances, behavioral stress

## Abstract

Mitochondrial dysfunction is a key factor in the pathophysiology of major depressive disorder (MDD) and treatment-resistant depression (TRD), connecting oxidative stress, neuroinflammation, and reduced neuroplasticity. Physical exercise induces specific mitochondrial changes linked to improvements in mental health. The aim of this paper was to examine emerging evidence regarding the effects of physical exercise on mitochondrial function and treatment-resistant depression, highlighting the clinical importance of the use of mitochondrial biomarkers to personalize exercise prescriptions for patients with depression, particularly those who cannot tolerate standard treatments. Physical exercise improves mitochondrial function, enhances biogenesis and neuroplasticity, and decreases oxidative stress and neuroinflammation. Essential signaling pathways, including brain-derived neurotrophic factor, AMP-activated protein kinase, active peroxisome proliferator-activated receptor-γ coactivator-1α, and Ca^2+^/calmodulin-dependent protein kinase, support these effects. Most studies have concentrated on the impact of low- and moderate-intensity aerobic exercise on general health. However, new evidence suggests that resistance exercise and high-intensity interval training also promote healthy mitochondrial adaptations, although the specific exercise intensity required to achieve this goal remains to be determined. There is strong evidence that exercise is an effective treatment for MDD, particularly for TRD, by promoting specific mitochondrial adaptations. However, key gaps remain in our understanding of the optimal exercise dose and which patient subgroups are most likely to benefit from it (Graphical Abstract).

## 1. Introduction

Major depressive disorder (MDD) is a serious mental health condition characterized by persistent low mood, loss of interest or pleasure in activities, and significant impairment in daily functioning, affecting how a person feels, thinks, and behaves [1]. MDD affects approximately 5% of the adult population worldwide, with 30% of them developing treatment-resistant depression (TRD), which is characterized by an inadequate response to two or more antidepressant treatments or therapies [2]. This means that many people cannot tolerate or do not respond to standard pharmacotherapies, leaving clinicians with few options for treating this health problem. Some critical consequences of MDD and TRD include poor quality of life [3], high healthcare costs, and lost productivity, with annual expenses in the U.S. alone estimated to be between $29 billion and $48 billion [4]. Additionally, there is an increased rate of suicide attempts and self-harming behaviors [5,6]. This situation makes MDD a leading cause of disability worldwide [7]. Nevertheless, physical exercise has been shown to protect against MDD and improve quality of life [8]. This protection is associated with improvements in skeletal muscle and neuronal mitochondrial function [9,10]. Exercise is low-cost, broadly accessible, and can be combined with medications and psychotherapy, offering a route for patients who cannot tolerate higher drug doses or polypharmacy. However, there is no clear guidance on which exercise modality or optimal dose could help to improve mitochondrial dysfunction in TRD. Conversely, prolonged periods of sedentarism have been associated with mitochondrial dysfunction and depressive symptoms [11]. Mitochondrial dysfunction is here defined as the inability of mitochondria to efficiently generate ATP through oxidative phosphorylation in response to cellular energy demands. This dysfunction results from various mitochondrial disturbances, some of which are related to a sedentary lifestyle and, in opposition, physical exercise.

This article highlights and integrates the biochemical mechanisms of neuronal mitochondrial dysfunction caused by systemic stress, proposing that a dose- and personalized physical exercise plan should be integrated into pharmacological and psychological therapies. This integrative plan has significant protective effects to improve mitochondrial function and reduce the symptoms of MDD, particularly in treatment-resistant patients.

## 2. Pathophysiology of Major Depressive Disorder

The biochemical mechanism of depressive disorder has been explained elsewhere [12,13]. These studies strongly suggest that the development of MDD, as well as mood and anxiety disorders, is linked to abnormal brain function [14,15,16,17], hyperactivation of the hypothalamic–pituitary–adrenal axis, severe systemic inflammation, mitochondrial dysfunction, and elevated levels of reactive oxygen species (ROS) [18,19]. Increased levels of inflammatory cytokines and ROS in cerebrospinal fluid interfere with mitochondrial DNA homeostasis, reduce the efficiency of oxidative phosphorylation in the respiratory chain, and hinder mitochondrial biogenesis [18,20,21].

**Figure 1:**
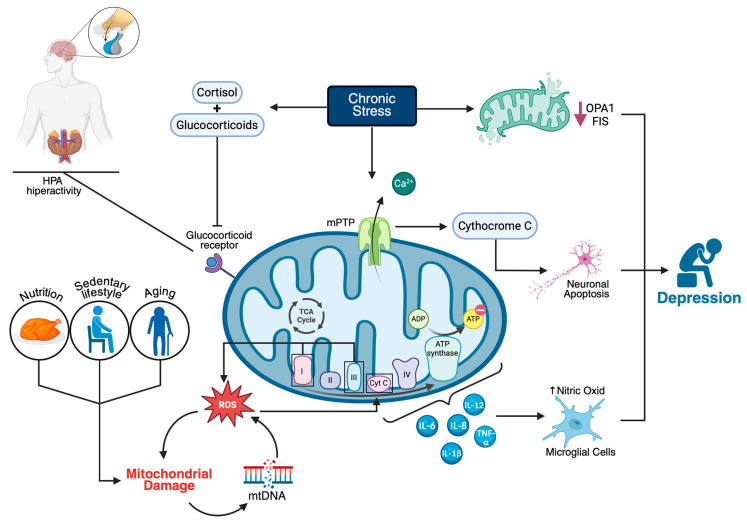
Mitochondrial dysfunction as a central mechanism in the pathophysiology of major depressive disorder (MDD). Chronic stress activates the hypothalamic–pituitary–adrenal (HPA) axis, leading to increased glucocorticoid and cortisol levels, which, through glucocorticoid receptor signaling, contribute to mitochondrial dysfunction. This process is exacerbated by factors such as aging, a sedentary lifestyle, and poor nutrition. Mitochondrial impairment is characterized by increased reactive oxygen species (ROS) in complexes I and III, damage to mitochondrial DNA (mtDNA), altered ATP synthesis, and elevated levels of proinflammatory cytokines (e.g., IL-6, IL-8, IL-12, IL-1β, TNF-α). Chronic stress reduces mitochondrial fusion proteins (OPA1 and FIS), promotes calcium overload and mitochondrial permeability transition pore (mPTP) opening, leading to cytochrome c release and neuronal apoptosis. These cellular alterations disrupt neurotransmission and neuroplasticity, ultimately contributing to the development of depressive symptoms.

The production of ATP through oxidative phosphorylation is closely linked to the flow of electrons along the inner mitochondrial membrane. However, in individuals with MDD, mitochondrial dysfunction is usually present [22,23], reflected by a proinflammatory profile and elevated levels of cytokines (e.g., interleukin-6, interleukin-8, interleukin-12, IL-1β, and TNF-α) [24,25]. Cytokines damage the electron transport chain (ETC) machinery and promote the release of nitric oxide from microglia, which interferes with cytochrome C oxidase (Complex IV) [24], decreases ATP production, and disrupts neuronal functions vital for mood regulation, such as neurotransmitter release and synaptic plasticity [26]. In animal models of depression, elevated ROS levels are linked to increased rates of mitochondrial DNA (mtDNA) mutations and deletions [27]. Moreover, damaged mtDNA initiates a vicious cycle in which faulty mitochondria produce more ROS, thereby worsening cellular stress and cell dysfunction (Figure 1). Unlike nuclear DNA, mtDNA lacks protective structures, such as histones, which increase its vulnerability to stress damage [28]. A mouse model of human depression and insomnia revealed damage to mitochondrial autophagy, decreased synthesis and secretion of melatonin, and elevated levels of IL-1β, NF-κB, Pink1, and Parkin in the pineal gland [18].

Another pathway of mitochondrial impairment, which has not yet been explored in the context of MDD and may be relevant, involves mutations in the putative kinase 1/Parkin E3 ubiquitin-protein ligase (PINK1/Parkin) genes or their deregulated expression. The PINK1/Parkin pathway is responsible for removing damaged mitochondria in hippocampal neurons [29,30], thus protecting against mitochondrial damage and reducing oxidative stress, both of which are essential for effective neurotrophic signaling. Li et al. (2025) reported increased levels of Pink1/Parkin expression alongside decreased levels of BDNF, Beclin 1, and BCL2 interacting protein 3 expression, a pattern linked to reduced autophagy in damaged pineal gland cells [18].

On the other hand, while selective serotonin reuptake inhibitors (SSRIs) are generally the most common first-line treatments for major depressive disorder (MDD), a significant percentage of patients fail to improve symptoms and instead progress to TRD [31]. Several hypotheses suggest that TRD may originate from severe mitochondrial dysregulation, neuroinflammation, and epigenetic changes that traditional pharmacotherapy does not effectively address [17,32]. Other reasons for pharmacological failure include the fact that each individual reacts differently to treatments; this is why metabolomic and genomic analyses are currently recommended to determine individual sensitivity to treatments. For example, Bhattacharyya et al. (2025) recently reported contrasting differences in the blood concentrations of several neuronal signaling markers in patients with TRD due to the chronic effects of different SSRIs [33].

## 3. Does Chronic Stress Induce Mitochondrial Dysfunction in Patients with Depressive Disorders?

Chronic stress is closely associated with neuronal mitochondrial dysfunction and depressive disorders. Animal models have shown that depression-like behaviors increase ROS levels in neuronal mitochondria and disrupt cellular signaling in the hippocampus and prefrontal cortex [34,35,36,37]. Treatments that restore mitochondrial function, such as mitochondrial transplantation, may help reverse these symptoms (Table 1).

### 3.1. Mitochondrial Dysfunction

Mitochondria are vital organelles involved in various metabolic processes, including energy generation, the biosynthesis of macromolecules, maintaining redox balance, regulating calcium homeostasis, managing cellular waste, and regulating apoptosis. Mitochondrial dysfunction disrupts these processes, leading to pathophysiological issues such as increased ROS production, mitochondrial DNA damage, tissue inflammation, decreased biogenesis, and impaired neuromuscular signaling [38,39], which contribute to the neurophysiological disturbances observed in mood disorders, anxiety, and MDD [22]. This phenomenon occurs in both skeletal muscle and neurons [40,41].

As described above, mitochondrial dysfunction is increasingly recognized as a factor associated with the development of depression. While current research does not prove that mitochondrial dysfunction directly causes depression, there is strong evidence that it contributes to depressive symptoms and that improving mitochondrial function can alleviate these symptoms. Research in both animal models and humans has shown that mitochondrial dysfunction disrupts neurotransmission and neuroplasticity, contributing to depressive symptoms [14,42]. Restoring mitochondrial function, whether through mitochondrial transplantation or exercise, restores mitochondrial function and decreases depressive symptoms [9,35,43].

Associations between chronic psychological stress and mitochondrial alterations have been shown in both human and animal studies [44]. Chronic stress induces morphological changes, including fragmented mitochondria, decreased cristae density, and the downregulation of mitochondrial fusion proteins (FIS1 and OPA1), both in vitro and in rodent models [45,46]. Furthermore, prolonged psychological stress and depression result in chronically elevated cortisol and glucocorticoid levels, which deactivate mineralocorticoid receptors and glucocorticoid receptors, leading to mitochondrial dysfunction and hyperactivity in the hypothalamic–pituitary–adrenal axis [47,48,49].

Another critical process involved in chronic depression is the disturbance of circulating mitochondrial DNA-related microRNAs and mitochondrial calcium deregulation [50]. In patients with depression, four weeks of selective serotonin reuptake inhibitor (SSRI) treatment decreases the levels of circulating mitochondrial DNA and related microRNAs (miR-6068 and miR-4708-3p) and depressive states [50], indicating that mitochondrial damage is linked to depressive states. Chronic stress or inflammatory conditions that lead to calcium overload can disturb the opening of the mitochondrial permeability transition pore and the release of proapoptotic factors such as cytochrome C [51]. Ongoing apoptosis of neuronal cells in the hippocampus and prefrontal cortex, regions vital for mood regulation, may considerably contribute to depression [52]. Additionally, lower brain energy metabolism, as measured by phosphocreatine levels (a marker of mitochondrial function), is correlated with higher depression scores in adolescents [53].

In this context, several therapeutic interventions have been proposed to enhance mitochondrial function and decrease MDD symptoms (Table 2).

### 3.2. Mitochondrial Biogenesis

Mitochondrial biogenesis, the process by which cells increase their mitochondrial mass, is regulated mainly by active peroxisome proliferator-activated receptor-γ coactivator-1α (PGC-1α) [26] and is enhanced by physical exercise [55]. PGC-1α collaborates with nuclear respiratory factor 1 and nuclear respiratory factor 2 to regulate the transcription of nuclear-encoded mitochondrial proteins [56], including Cox10, Cox15, and the β-subunit of ATP synthase. These proteins are utilized for oxidative metabolism, ETC assembly, and mitochondrial ATP synthesis. AMP-activated protein kinase (AMPK) senses the energy status of a cell. It can be activated when there are low levels of ATP, a condition often found in response to exercise [57]. AMPK is activated during exercise and phosphorylates downstream substrates, such as PGC-1α, thereby increasing the activity of genes related to mitochondria and promoting mitochondrial biogenesis [58].

Additionally, the Ca^2+^/calmodulin-dependent protein kinase II pathway is activated by increased intracellular Ca^2+^ during exercise, resulting in the phosphorylation of cAMP response element-binding protein (CREB) and subsequent transcription of BDNF [59]. This signaling improves mitochondrial biogenesis and synaptic plasticity, as well as neuroprotection, which have positive effects on cognitive function through impacts on neurogenesis and the remodeling of synaptic contacts, all of which are essential for managing stress and depression.

### 3.3. Oxidative Stress and Mitochondrial Dynamics

Mitochondria play a central role in both the production of ROS and detoxification; these biochemical mechanisms are well-documented elsewhere. Briefly, during oxidative phosphorylation, the ETC transfers electrons from NADH and FADH_2_ to oxygen, producing H_2_O. A small fraction of these electrons prematurely reduces O_2_ to superoxide (O_2_^−^), which is then enzymatically converted to hydrogen peroxide (H_2_O_2_) and, via metal-catalyzed reactions, to the highly reactive hydroxyl radical (•OH). Oxidative stress occurs when ROS production surpasses the antioxidant defenses, leading to damage of lipids, proteins, and mitochondrial DNA, and disrupting signaling and energy production [60,61]. Mitochondrial ROS mainly originate from complexes I (NADH: ubiquinone oxidoreductase) and III (ubiquinol: cytochrome C oxidoreductase), both of which produce superoxide [60,61,62,63]. Among ROS, the hydroxyl radical (•OH), produced in the mitochondrial matrix, is the most inherently harmful because it reacts almost at diffusion-controlled rates with nearly all biomolecules and lacks specific enzymatic scavengers. It mainly forms from H_2_O_2_ through Fenton and Haber-Weiss reactions involving redox-active iron or copper, causing severe damage at the site of its formation, including mitochondrial DNA and inner-membrane polyunsaturated lipids. Peroxynitrite (ONOO^−^), formed from the near diffusion-limited reaction of superoxide with nitric oxide, is also highly cytotoxic; it oxidizes and nitrates proteins, inactivates enzymes, especially those with iron–sulfur clusters, and initiates lipid peroxidation, further impairing mitochondrial function [64]. By contrast, superoxide itself is short-lived and largely compartmentalized, and H_2_O_2_, although less reactive, becomes dangerous when its local concentration exceeds the capacity of peroxidases [60,61]. In this paper, the importance of ROS lies in its role as a source of mitochondrial pathologies, where MDD and TRD are implicated.

Neuronal cells are highly vulnerable to oxidative damage because they depend on high and efficient energy production by mitochondria. Antioxidant defenses in mammals against the damaging effects of ROS during exercise and for the maintenance of redox homeostasis include the activities of enzymatic antioxidants such as superoxide dismutase (SOD), catalase (CAT), and glutathione peroxidase (GSH-Px) [65].

SOD, CAT, and GSH-Px are the most potent enzymatic antioxidant defenses against oxidative stress and neuronal oxidative damage. The first line of defense includes SOD, which catalyzes the dismutation of superoxide anions to hydrogen peroxide; then, CAT and GSH-Px breakdown hydrogen peroxide into water and molecular oxygen. Table 3 illustrates that mitochondrial dynamics encompass two essential processes: fusion and fission, which are crucial not only for maintaining mitochondrial function but also for preserving morphology, distribution within neurons, and repairing damaged mtDNA. The above-described process is controlled by MFN1 and OPA1, which, through DRP1 and FIS1, regulate this process. This coordinated activity ensures proper mitochondrial function, distribution, and quality control, thereby facilitating optimal neuron health. Protein dysregulation has been noted to be involved in neurodegeneration and impairments in neuronal health [66]. The normalization of mitochondrial dynamics prevents neuronal degeneration and cognitive impairments [38]. Exercise-induced regulation of fusion and fission proteins (dynamin-related proteins) in the direction of control maintains a healthy mitochondrial network [67].

### 3.4. Neuroplasticity and Depression

Neuroplasticity refers to the brain’s ability to adapt to stressors, encode experiences, and recover from physical and metabolic injuries [68]. Neuronal resilience and plasticity depend on proper glial function, particularly that of astrocytes and microglia, which also require intact mitochondrial function for neuronal metabolism and the regulation of the neuroinflammatory stress environment [69,70,71].

Depression and chronic stress are associated with disruptions in neuronal signaling pathways and synaptic plasticity, particularly in brain regions such as the prefrontal cortex and hippocampus [49,72]; where mitochondria play a central role in holding neuroplasticity by supplying the energy (ATP) needed for neurite outgrowth, synapse formation, and long-term potentiation [73]. A high concentration of mitochondria is present in presynaptic terminals, supplying ATP for synaptic vesicle recycling and maintaining the ionic gradients needed for excitability and synaptic transmission [74,75]. Mitochondrial dysfunction caused by chronic stress can drive glial cells toward a proinflammatory state, disrupting synaptic homeostasis and leading to synaptic atrophy, as observed in depressive disorders [76]. Chronic stress impairs mitochondrial energy production in astrocytes, leading to reduced glutamate removal from synapses and increased excitotoxicity, particularly in the prefrontal cortex and hippocampus [77,78,79,80,81]; this ultimately hinders the survival and plasticity of neurons, contributing to the development of depressive symptoms [49]. Rial et al. (2016) reported that in depression, there is a notable decline in astrocyte density and function, accompanied by increased microglial activation in frontolimbic regions, which may contribute to synaptic damage [82].

In contrast, physical exercise significantly enhances synaptic plasticity through various structural and molecular mechanisms, benefiting cognitive functions and facilitating recovery from neurological conditions. Specifically, an 8-week treadmill exercise program increased excitability and synaptic transmission, as well as short- and long-term potentiation, in the hippocampus of 6-month-old APP/PS1 mice. This improvement was correlated with an increase in the number of synaptic structures [83]. In addition, exercise increases synaptic plasticity by promoting the expression of CaMK2a and CYFIP1 through the upregulation of CaMK2a, both of which are involved in dendritic remodeling and synaptic strength [84]. Additionally, the activity of AMPAR following aerobic exercise increases the caveolin-1/VEGF signaling pathway, contributing to the enhancement of synaptic plasticity [85].

Given that altered neuroplasticity and mitochondrial function are connected to the development of depression, identifying new treatment options, such as physical exercise, could serve as an effective intervention to reduce mitochondrial damage and neuroinflammation, thereby restoring neuroplasticity and decreasing mood disorders.

### 3.5. Role of BDNF

BDNF is a neurotrophin essential for neuronal survival, synaptic plasticity, and brain function. It is highly expressed in the brain, particularly in the cortex, hippocampus, basal forebrain, and other regions crucial for learning and memory, and may also function as a protective mechanism against central nervous system diseases, such as depression [86]. Neuronal BDNF levels increase via N-methyl-d-aspartate receptor activation and bind to the tropomyosin receptor kinase B, localized to mitochondria, activating protein kinase A signaling and phosphorylation of proteins Drp1 and Miro-2 [87]. This cascade enhances mitochondrial fusion, trafficking, and content in neurons, leading to increased mitochondrial respiration and ATP production [87].

Decreased BDNF levels, in most brain regions observed during depressive conditions, contribute to the pathogenesis of mood disorders through various interactions with neurotransmitter systems [88]. Conversely, high BDNF levels are strongly correlated with positive outcomes, as this factor promotes neuronal survival, differentiation, and plasticity pathways through the modulation of intracellular calcium and gene transcription related to mitochondrial respiration [87,89].

The synthesis of BDNF is one of the significant effects of exercise, helping in the formation and remodeling of synaptic connections, stimulating the production of new neurons, and reinforcing existing connections by activating genes that promote the growth and stabilization of brain cells. During exercise, increases in β-hydroxybutyrate and lactate stimulate the synthesis of BDNF, as both molecules cross the blood–brain barrier and induce its expression within the brain [90,91]. Animal and in vitro studies have shown that β-hydroxybutyrate increases BDNF expression in hippocampal neurons by activating the cAMP/PKA/p-CREB signaling pathway [92] and through tropomyosin receptor kinase B [91], thereby enhancing neuronal activity. In vitro studies also indicate that lactate promotes hippocampal BDNF expression. During high-intensity interval training (HIIT), blood lactate concentrations increase significantly [93].

### 3.6. Nutrients in Individuals with Depressive Disorders

Vitamins B6 and B12, together with tryptophan, maintain mitochondrial function, and vitamins B6 and B12, together with tryptophan, maintain mitochondrial function and prevent neuronal damage. Vitamin B6 can antagonize mitochondrial dysfunction and oxidative stress in the hippocampus, regardless of stress conditions, by modulating key signaling pathways (p-JNK/Nrf-2/NF-κB) and restoring synaptic protein levels [94]; this could help reduce depressive disorders. Similarly, Didangelos et al. showed that vitamin B12 supplementation in diabetic rats reduces neuronal apoptosis and degeneration, restoring neurotrophic support and enhancing synaptic plasticity [95], which highlights the importance of this nutrient in maintaining mitochondrial health and overall neuronal integrity under metabolic stress conditions. Tryptophan metabolism, particularly through the kynurenine pathway, is closely linked to brain health and mitochondrial function in neural cells [96]. Tryptophan deficiency may reduce the viability of mitochondria in neural cells, specifically through the disruption of the tryptophan-kynurenine pathway [97]. The altered metabolism of tryptophan under conditions of neuroinflammation or stress reroutes more than normal amounts toward kynurenine owing to increased activity of enzymes such as indoleamine 2,3-dioxygenase. This enzyme catalyzes the first rate-limiting step of tryptophan catabolism, which is part of its route to form serotonin and melatonin [35]. This shift is associated with a decrease in mitochondrial membrane potential and ATP production, along with an increase in neurotoxic metabolites, all of which contribute to mitochondrial dysfunction and neuronal damage, as observed in animal models of stress and brain injury. Conversely, tryptophan supplementation has been shown to improve mitochondrial function, increase antioxidant capacity, and reduce inflammation and cell death in animal models subjected to stress [98]. Therefore, a deficiency of these nutrients could result in mitochondrial dysfunction and increased oxidative stress. Although direct evidence of mitochondrial damage from low blood levels of B6, B12, and tryptophan is limited, these results suggest that low blood concentrations of these nutrients may impair mitochondrial function, especially under chronic stress, because they act as neuroprotective agents by reducing oxidative stress, inflammation, and apoptosis, factors often linked to mitochondrial dysfunction in neurodegenerative and metabolic diseases. Overall, these findings emphasize the potential of B vitamins and tryptophan as therapeutic supplements to support mitochondrial health and protect the brain against neurodegeneration and depressive disorders.

## 4. Does Physical Exercise Protect Against Depressive Disorder?

Exercise is an intervention widely associated with various health benefits, including improved cardiovascular function and neuroprotective properties for several psychiatric and neurological disorders [99]. The antidepressant effects of exercise are also well established [100]; however, it has only recently been linked to mitochondrial biogenesis. This relationship becomes more relevant in TRD populations, as the traditional pharmacological approach often presents suboptimal and sometimes unstable relief. Hence, the mitochondria-centered approach is a valuable methodology to optimize the prescription of exercise for mental health-related disorders where conventional treatments do not work.

Regular physical exercise is a potent nondrug therapy capable of preventing and reducing neuronal inflammation through several pathways: lowering the levels of proinflammatory cytokines (e.g., TNF-α and IL-6), increasing the expression of anti-inflammatory cytokines (e.g., IL-10 and IL-35), enhancing antioxidant capacity, and increasing BDNF levels [101,102]. These effects contribute to decreased oxidative damage in neuronal tissues, improved mood and brain health, reduced neuropathic pain, and enhanced cognitive and motor skills. Specifically, exercise increases the levels of neurotrophic factors (e.g., BDNF), which are essential for neuronal survival, development, and plasticity [103]. Furthermore, BDNF promotes homeostatic mitochondrial turnover, particularly by regulating the expression of proteins involved in mitochondrial fusion and fission [104,105]. In this context, endurance exercise increases mitochondrial fusion and fission (autophagy/mitophagy) processes in the brain, thereby increasing mitochondrial turnover and maintaining a healthy cellular environment [105]. As a result, the combined effect of all these pathways decreases neuroinflammation, enhances synaptic plasticity and mitochondrial function, and helps to reduce depression symptoms.

A cross-sectional study found that men with depressive symptoms exhibited significantly lower serum testosterone levels compared to non-depressed controls [106]. Another mouse model study found parallel increases in both testosterone and BDNF following aerobic exercise training [107]. Animal studies indicate that testosterone can modulate BDNF levels in the brain in a dose-specific manner, influencing cognitive and neuroprotective functions [108]. However, a direct association between serum testosterone levels and BDNF in individuals with depressive symptoms has not been established.

### 4.1. Sedentarism and Depression

Sedentary behavior is characterized by extended periods of physical inactivity [11] and is associated with several health problems, including muscle atrophy, neurodegenerative diseases, mitochondrial dysfunction, and depression symptoms. These problems are influenced by several factors, such as age, mobility limitations, sleep impairments, pain, anxiety, and social isolation [109]. Adults who spend 50% or more of their leisure time in sedentary activities experience more frequent depression and anxiety symptoms [110]. Endrighi et al. (2016) reported that two weeks of free-living sedentary time can lead to mood disturbances in healthy adults [111]. Additionally, Schuch et al. (2017) reported that individuals with MDD perform less physical activity and are more involved in sedentary behavior [112].

Systematic physical exercise programs in populations with MDD have been shown to decrease depressive symptoms and enhance overall mental health, and improvements associated with positive changes in mitochondrial function [8,113] suggest that replacing sedentary activities with light, moderate, or vigorous physical activity combined with adequate sleep may significantly reduce symptoms of depression [114].

### 4.2. Sedentarism and Mitochondrial Dysfunction

Sedentarism, or prolonged physical inactivity, has been shown to impair muscle mitochondrial function [115]. Multiple studies in animals and humans have confirmed that a sedentary lifestyle reduces mitochondrial capacity, increases oxidative stress, and impairs metabolic flexibility [79,116]. Figueiredo et al. (2009) reported that, compared with their active counterparts, lifelong sedentary behavior in mice results in a decline in skeletal muscle mitochondrial function, with increased oxidative damage to mitochondrial biomolecules and a greater loss of muscle mass [117]. In rats, a short-term switch from an active to a sedentary routine results in a rapid increase in oxidative stress, a decrease in the activity of antioxidant enzymes (superoxide dismutase, catalase, and glutathione peroxidase), and an increase in the levels of oxidative damage markers (e.g., protein oxidation and 4-hydroxynonenal) in muscle tissue [118].

**Figure 2:**
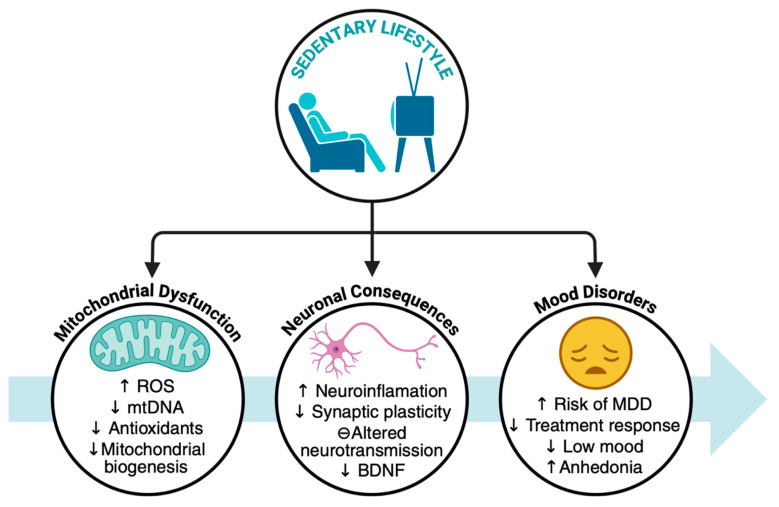
Sedentary lifestyle as a contributing factor to mitochondrial dysfunction, neuronal alteration, and mood disorders. BDNF: Brain-derived neurotrophic factor, MDD: Major depressive disorder, mtDNA: Mitochondrial DNA, ROS: Reactive oxygen species. The arrows mean increase (↑) and decrease (↓).

Studies of in vitro (neuronal cells of a mouse model) sedentary states (such as chronic sleep fragmentation, which often accompanies sedentary lifestyles) have revealed a reduced number of mitochondria, impaired mitochondrial respiratory chain components, and decreased mitochondrial DNA in critical brain regions for cognition. These changes are associated with disrupted mitochondrial biogenesis signaling pathways and worsened cognitive function [119]. Furthermore, in a mouse model of mitochondrial disorders, sedentary behavior was associated with pronounced cerebellar dysfunction and mitochondrial deficiency [41] (Figure 2).

### 4.3. Effects of Different Types of Exercise on Mitochondrial Function and Depression

Physical exercise is increasingly recognized for its role in enhancing both mitochondrial function and mental health, particularly in the treatment of depression. Different types of exercise, as mentioned below, influence biological and psychological outcomes in distinct ways.

#### 4.3.1. Aerobic Exercise

Aerobic exercise training (such as running, cycling, or swimming at moderate intensity) is well documented to enhance brain mitochondrial function and mitochondrial oxidative capacity. Studies in a mouse model of Alzheimer’s disease have shown that aerobic exercise upregulates CD38 expression in astrocytes, facilitating the CD38-mediated transfer of healthy mitochondria from astrocytes to neurons [9]. Additionally, it increases PGC-1α, SIRT1, and the expression of citrate synthase, as well as microRNA, within hippocampal astrocytes and across most brain regions, promoting the transfer of healthy mitochondria from astrocytes to neurons and regulating mitochondrial proteostasis, which in turn increases brain mitochondrial biogenesis [10]. In old Wistar rats, aerobic exercise increased the expression of the OPA1 gene in the hippocampus, reduced the expression of the Drp1 gene, modulated mitochondrial fusion and fission processes, and improved spatial learning and memory performance [120]. Fernández et al. (2020) reported that combined exercise training (aerobic and resistance) increases mitochondrial complex V activity in the brains of a mouse model [41]; however, animal studies are not always transferable to humans. While there are conserved elements of mitochondrial metabolism across species [121], significant differences exist, particularly in how metabolic dysfunctions manifest and are regulated [122].

#### 4.3.2. Resistance Exercise

Resistance training has long been a subject of discussion regarding mitochondrial adaptations. Animal studies (in rats with sporadic inclusion body myositis) have shown that resistance training reduces damage and skeletal muscle atrophy, increases mitochondrial biogenesis, and decreases amyloid-beta protein (Aβ) accumulation [123,124]. However, human studies have shown little to no effect on mitochondrial function [125]. Twelve weeks of resistance training increased muscle strength and muscle oxidative capacity, increasing the proportion of neural cell adhesion molecule-positive satellite cells and restoring normal mitochondrial function in patients with sporadic mitochondrial DNA mutations in skeletal muscle [126]. A 21-day resistance training program following 10 days of muscle disuse increases muscle mass, function, mitochondrial activity, and biogenesis [127]. Twelve weeks of resistance exercise training increased the mitochondrial respiratory capacity and muscle strength without inducing mitochondrial biogenesis in young, healthy men [128]. Six weeks of high-volume resistance training decreased citrate synthase activity by 24% and reduced the concentrations of actin and myosin proteins in the muscle fibers of college resistance-trained individuals [129]. Thus, the findings regarding the benefits of resistance training on mitochondrial function are inconsistent.

#### 4.3.3. High-Intensity Interval Training

HIIT involves short bouts of near-maximal or maximal effort interspersed with rest or low-intensity exercise periods. HIIT increased lactate levels and promoted hippocampal MCT1/4 and BDNF expression. HIIT can induce skeletal mitochondrial adaptations (increased mitochondrial content, citrate synthase, and cytochrome C oxidase in skeletal muscle) [130,131,132]. HIIT stimulates BDNF expression in the hippocampus, enhancing brain function in a mouse model [93]. One week of HIIT increased BDNF, doublecortin, and voltage-dependent anion-selective channel protein 2 in a mouse model. Nevertheless, it decreased mitochondrial superoxide dismutase 2 content in the hippocampus, with no changes in redox status [133].

**Figure 3:**
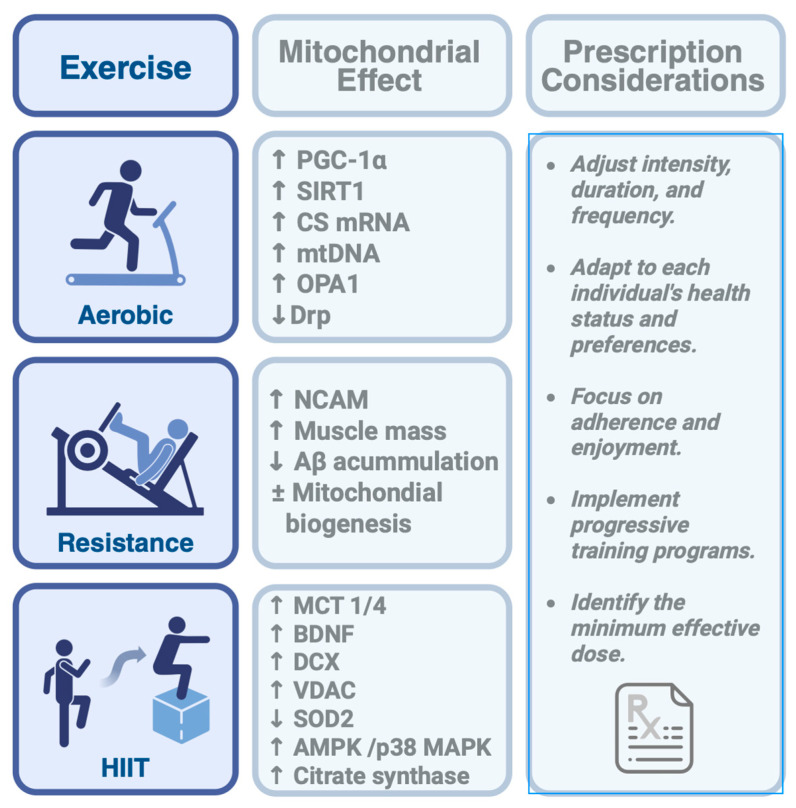
Mitochondrial adaptations and prescription considerations across different types of physical exercise. Aerobic, resistance, and high-intensity interval training (HIIT) each promote specific mitochondrial responses. Aβ: amyloid-beta protein, AMPK: AMP-activated protein kinase, BDNF: Brain-derived neurotrophic factor, CS mRNA: Citrate synthase microRNA, DCX: Doublecortin, MCT: Monocarboxylate transporters, mtDNA: Mitochondrial DNA, NCAM: Neural cell adhesion molecule, OPA1: Optic Atrophy 1, PGC-1α: Active peroxisome proliferator-activated receptor-γ coactivator-1α, p38 MAPK: p38 mitogen-activated protein kinase, SIRT1: Silent Information Regulator T1, SOD2: Superoxide dismutase 2, VDAC: Voltage-dependent anion-selective channel protein 2. The arrows mean increase (↑) and decrease (↓).

The high-energy stress associated with HIIT strongly activates either AMPK or p38 MAPK [134]. Both kinases converge on PGC-1α, leading to increased mitochondrial biogenesis. Studies on rodent models and humans have repeatedly shown that HIIT interventions increase the activity of citrate synthase, an important marker of mitochondrial content [135]. Although HIIT is unlikely to be available to all patients, the significant mitochondrial adaptations promise to represent a time-efficient strategy for augmenting the current treatment landscape for mental health (Figure 3).

### 4.4. Duration, Frequency, and Intensity of Exercise

As mentioned above, moderate- and high-intensity physical exercise induces significant changes in mitochondrial metabolism; however, there is no single type of exercise, intensity, volume, or frequency that works for everyone, as these factors vary depending on the disease and particular needs [136]. Nevertheless, current epidemiological, systematic, and meta-analyses indicate that the effectiveness of physical exercise as a treatment for depression depends on the duration and intensity of the exercise programs. Moderate- and high-intensity exercise interventions lasting more than 8 weeks, with exercise sessions of ≥150 min per week or more, are most effective, reducing depression symptoms by 20% to 60% [137,138,139].

Additionally, factors such as age, baseline fitness, and the presence of chronic diseases can influence responses to exercise [140]. For cases of anxiety and depression, exercise prescriptions must consider the psychological and clinical aspects of the patient, the environment, and personal preferences, which could be crucial in maximizing the antidepressant and anxiolytic benefits of exercise. Furthermore, patients with TRD may experience motivational impediments and have a limited active range of motion due to long-standing depression symptoms. Therefore, structured, progressive exercise interventions are recommended to focus on adherence, enjoyment, and psychological support. Future studies should aim to identify the minimum effective dose that improves mitochondrial function in these patients.

## 5. Summary

This review examines the biochemical and clinical evidence that exercise-induced mitochondrial adaptation can reduce the symptoms of MDD, particularly in treatment-resistant patients. Such evidence may help explain the consistent link between oxidative stress, systemic inflammation, impaired mitochondrial function, chronic psychological stress, and MDD. All these factors impair neuroplasticity and worsen psychiatric prognosis. By focusing on mitochondrial dysfunction, this review reinterprets TRD as a disorder with complex metabolic and inflammatory roots rather than just a neurochemical imbalance, offering new targets for the treatment and recovery of those with chronic depression whose quality of life has been severely affected. The potential for exercise to modulate neuronal metabolism, especially mitochondrial function, in treating psychiatric disorders is promising as a therapeutic approach for MDD; this is especially important given the diversity of depression and the varying ways in which treatment resistance presents. Furthermore, physical exercise is a relatively affordable and accessible option for many communities and can be prescribed alongside drug therapy for patients with TRD.

Mitochondrial biogenesis and antioxidant defenses are promoted by physical exercise through key signaling pathways, including BDNF, AMPK, PGC-1α, and CaMK, which act to counteract the biochemical and cellular insults of stress and depression. Interim studies and clinical trials involving populations with TRD have shown that physical exercise lessens depression symptoms in these patients, while also enhancing mitochondrial function, increasing energy capacity, and boosting antioxidant enzyme activity.

Aerobic, resistance, or high-intensity interval training interventions are effective methods to improve MDD and TRD; however, because of the wide variety of study approaches and different types of physical exercises used, we only have general recommendations and no precise data on the ideal intensity, volume, or type of exercise for treating this condition. As a result, each case remains unique. Considering the evidence presented here, we propose that exercise should be viewed not only as an adjunct but also as a fundamental therapeutic principle for patients with TRD owing to its extensive metabolic, anti-inflammatory, and brain health benefits. Exercise may have a synergistic effect when combined with current pharmacotherapies and psychotherapies. Physicians should encourage their patients to incorporate regular exercise into their daily routines. However, patients with TRD often have lower motivation because of the disease itself. Hence, such structured programs will encompass behavioral support, social interaction, and regular follow-up to sustain adherence and realize maximal long-term benefits.

Future research should include more extensive, well-controlled randomized controlled trials involving treatment-resistant depression patients, incorporating clearly defined mitochondrial biomarkers and standardized exercise protocols to determine causality and refine exercise dosage. While animal models have proven useful in identifying exercise-mediated adaptations, their relevance to humans remains limited. Therefore, there is a strong need for direct studies in humans using either muscle biopsies or peripheral blood mononuclear cell samples. Additionally, innovative noninvasive or minimally invasive methods to assess central mitochondrial function should be developed and thoroughly validated to help identify early disease stages, assess severity, and monitor treatment effectiveness.

In summary, existing evidence indicates that exercise-induced mitochondrial adaptations are promising but underused therapeutic targets in TRD. Future research connecting basic science with clinical practice can help identify the best ways to harness the benefits of exercise to improve the lives of those with treatment-resistant depression.

## Figures and Tables

**Table 1:** Chronic Stress, Mitochondrial Dysfunction, and Depression-like Behaviors.

Model	Stress Factor	Intervention	Outcome	Citation
Male Wistar rats	Chronic behaviors stress	Mitochondrial transplantation	Restored mitochondrial function and increased ATP	[34,35,36]
Sprague–Dawley rats	Maternal separation	Antidepressants	Restores mitochondrial function and increases ATP	[34]
Adolescent cynomolgus monkeys	Chronic unpredictable mild stress	Chronic stress	Increased metabolic dysfunction and depression	[37]

**Table 2:** Proposed therapeutic interventions to enhance mitochondrial function and reduce depressive disorders or their severity.

Intervention	Model	Effect on Mitochondria	Effects on Depression-Like Behaviors	Citations
Mitochondrial transplantation	Murine	Restores function, increases ATP	Reduces depressive symptoms	[35,36]
Physical exercise	Murine	Normalizes mitochondrial activity	Improves mood, reduces symptoms	[43,54]
Herbal treatments (e.g., Sinisan)	Murine	Improves mitochondrial function	Alleviates depressive behaviors	[34]
Mirtazapine, paroxetine, or sertraline	Human	Improves mitochondrial function	Remission of MDD	[50]
Creatine supplementation	Human	Increases brain energy stores	Correlates with symptom reduction	[53]

**Table 3:** Mitochondrial dynamics of MFN1, OPA1, and DRP1.

Mitofusin 1 (MFN1) is found in the outer mitochondrial membrane and facilitates the fusion of neighboring mitochondria by connecting their outer membranes. It collaborates with MFN2 to preserve the integrity and function of the mitochondrial network. MFN1 is vital for maintaining connected mitochondria in neurons, which is crucial for energy distribution and calcium buffering in axons and dendrites.
Optic Atrophy 1 (OPA1) is a GTPase located in the inner mitochondrial membrane, responsible for fusion within the inner mitochondrial membrane (IMM) and cristae remodeling. It plays a key role in protecting mitochondrial structure and bioenergetics. In neurons, OPA1 is essential for synaptic function and neuroprotection. Abnormal regulation of OPA1 activity has been associated with optic atrophy and Parkinson’s disease.
Dynamin-Related Protein 1 (DRP1) is a GTPase that is vital for mitochondrial fission through constricting and dividing mitochondria at specific sites on the outer mitochondrial membrane. DRP1 is critical for distributing mitochondria in neurons, especially in axons and synapses, where energy demands are high. However, excessive activity of this protein can cause mitochondrial fragmentation, synaptic issues, and neurodegeneration, as observed in Alzheimer’s and Parkinson’s diseases.
Fission 1 Protein (FIS1) collaborates with DRP1 to promote mitochondrial fission. Its precise role in humans is debated, but it is believed to act as a receptor recruiting DRP1. In neurons, FIS1 plays a role in regulating mitochondrial size and distribution. Abnormal regulation of FIS1 is linked to mitochondrial dysfunction and cognitive decline in neurodegenerative diseases.

## Abbreviations

AMPAR	α-amino-3-hydroxy-5-methyl-4-isoxazolepropionic acid receptor
AMPK	AMP-activated protein kinase
ATP	Adenosine triphosphate
BDNF	Brain-derived neurotrophic factor
CAT	Catalase
CYFIP1	Cytoplasmic FMRP-associated protein 1
Cox	Cytochrome C oxidase
CREB	cAMP response element-binding protein
CS mRNA	Citrate synthase microRNA
DCX	Doublecortin
DNA	Deoxyribonucleic acid
DRP1	Dynamin-Related Protein 1
ETC	Electron transport chain
FIS1	Fission Protein 1
GSH-Px	Glutathione peroxidase
HIIT	High-intensity interval training
IDO	Indoleamine 2,3-dioxygenase
IL	Interleukin
IMM	Inner mitochondrial membrane
LTP	Long-term potentiation
MCT	Monocarboxylate transporters
MDD	Major depressive disorder
MFN1, 2	Mitofusin 1 and 2
mtDNA	Mitochondrial DNA
NCAM	neural cell adhesion molecule
NF-κB	Nuclear factor kappa B
OPA	Optic Atrophy
p-JNK	Protein JUN N-terminal kinases
p38 MAPK	p38 mitogen-activated protein kinase,
Parkin	Ubiquitin-protein ligase
PBMC	Peripheral blood mononuclear cell
PGC-1α	Active peroxisome proliferator-activated receptor-γ coactivator-1α
PINK1/Parkin	putative kinase 1/Parkin E3 ubiquitin-protein ligase
ROS	Reactive oxygen species
SIRT1	Silent Information Regulator T1
SOD	Superoxide dismutase
SSRIs	Selective serotonin reuptake inhibitors
TNF-α	Tumoral necrosis factor α
TRD	Treatment-resistant depression
VEGF	Vascular endothelial growth factor

## References

[1] Marx W., Penninx B.W.J.H., Solmi M., Furukawa T.A., Firth J., Carvalho A.F., Berk M. (2023). Major Depressive Disorder. Nat. Rev. Dis. Primers.

[2] World Health Organization Depressive Disorder (Depression). https://www.who.int/news-room/fact-sheets/detail/depression.

[3] Fekadu A., Wooderson S.C., Markopoulo K., Donaldson C., Papadopoulos A., Cleare A.J. (2009). What Happens to Patients with Treatment-Resistant Depression? A Systematic Review of Medium to Long Term Outcome Studies. J. Affect. Disord..

[4] Mrazek D.A., Hornberger J.C., Altar C.A., Degtiar I. (2014). A Review of the Clinical, Economic, and Societal Burden of Treatment-Resistant Depression: 1996–2013. Psychiatr. Serv..

[5] Gronemann F.H., Jørgensen M.B., Nordentoft M., Andersen P.K., Osler M. (2021). Treatment-Resistant Depression and Risk of All-Cause Mortality and Suicidality in Danish Patients with Major Depression. J. Psychiatr. Res..

[6] Reutfors J., Andersson T.M.-L., Brenner P., Brandt L., DiBernardo A., Li G., Hägg D., Wingård L., Bodén R. (2018). Mortality in Treatment-Resistant Unipolar Depression: A Register-Based Cohort Study in Sweden. J. Affect. Disord..

[7] Rush A.J., Trivedi M.H., Wisniewski S.R., Nierenberg A.A., Stewart J.W., Warden D., Niederehe G., Thase M.E., Lavori P.W., Lebowitz B.D. (2006). Acute and Longer-Term Outcomes in Depressed Outpatients Requiring One or Several Treatment Steps: A STAR*D Report. Am. J. Psychiatry.

[8] Philippot A., Dubois V., Lambrechts K., Grogna D., Robert A., Jonckheer U., Chakib W., Beine A., Bleyenheuft Y., De Volder A.G. (2022). Impact of Physical Exercise on Depression and Anxiety in Adolescent Inpatients: A Randomized Controlled Trial. J. Affect. Disord..

[9] Cai J., Chen Y., She Y., He X., Feng H., Sun H., Yin M., Gao J., Sheng C., Li Q. (2025). Aerobic Exercise Improves Astrocyte Mitochondrial Quality and Transfer to Neurons in a Mouse Model of Alzheimer’s Disease. Brain Pathol..

[10] Steiner J.L., Murphy E.A., McClellan J.L., Carmichael M.D., Davis J.M. (2011). Exercise Training Increases Mitochondrial Biogenesis in the Brain. J. Appl. Physiol..

[11] del Pozo Cruz B., Alfonso-Rosa R.M., McGregor D., Chastin S.F., Palarea-Albaladejo J., del Pozo Cruz J. (2020). Sedentary Behaviour Is Associated with Depression Symptoms: Compositional Data Analysis from a Representative Sample of 3233 US Adults and Older Adults Assessed with Accelerometers. J. Affect. Disord..

[12] Gulyaeva N.V. (2021). Stress-Associated Molecular and Cellular Hippocampal Mechanisms Common for Epilepsy and Comorbid Depressive Disorders. Biochemistry.

[13] Wang Y., Cai X., Ma Y., Yang Y., Pan C.-W., Zhu X., Ke C. (2024). Metabolomics on Depression: A Comparison of Clinical and Animal Research. J. Affect. Disord..

[14] Czarny P., Ziółkowska S., Kołodziej Ł., Watała C., Wigner-Jeziorska P., Bliźniewska-Kowalska K., Wachowska K., Gałecka M., Synowiec E., Gałecki P. (2023). Single-Nucleotide Polymorphisms in Genes Maintaining the Stability of Mitochondrial DNA Affect the Occurrence, Onset, Severity and Treatment of Major Depressive Disorder. Int. J. Mol. Sci..

[15] Gardner A., Johansson A., Wibom R., Nennesmo I., von Döbeln U., Hagenfeldt L., Hällström T. (2003). Alterations of Mitochondrial Function and Correlations with Personality Traits in Selected Major Depressive Disorder Patients. J. Affect. Disord..

[16] Katrenčíková B., Vaváková M., Paduchová Z., Nagyová Z., Garaiova I., Muchová J., Ďuračková Z., Trebatická J. (2021). Oxidative Stress Markers and Antioxidant Enzymes in Children and Adolescents with Depressive Disorder and Impact of Omega-3 Fatty Acids in Randomised Clinical Trial. Antioxidants.

[17] Ni P., Ma Y., Chung S. (2024). Mitochondrial Dysfunction in Psychiatric Disorders. Schizophr. Res..

[18] Li Z., Shu Y., Liu D., Xie S., Xian L., Luo J., Huang X., Jiang H. (2025). Pink1/Parkin Signaling Mediates Pineal Mitochondrial Autophagy Dysfunction and Its Biological Role in a Comorbid Rat Model of Depression and Insomnia. Brain Res. Bull..

[19] Picard M., Juster R.-P., McEwen B.S. (2014). Mitochondrial Allostatic Load Puts the “gluc” Back in Glucocorticoids. Nat. Rev. Endocrinol..

[20] Braz G.R.F., de Andrade Silva S.C., da Silva Pedroza A.A., de Lemos M.D., de Lima F.A., da Silva A.I., Lagranha C.J. (2020). Fluoxetine Administration in Juvenile Overfed Rats Improves Hypothalamic Mitochondrial Respiration and REDOX Status and Induces Mitochondrial Biogenesis Transcriptional Expression. Eur. J. Pharmacol..

[21] Gonçalves V.F., Mendes-Silva A.P., Koyama E., Vieira E., Kennedy J.L., Diniz B. (2021). Increased Levels of Circulating Cell-Free mtDNA in Plasma of Late Life Depression Subjects. J. Psychiatr. Res..

[22] Jiang M., Wang L., Sheng H. (2024). Mitochondria in Depression: The Dysfunction of Mitochondrial Energy Metabolism and Quality Control Systems. CNS Neurosci. Ther..

[23] Teranishi M., Ito M., Huang Z., Nishiyama Y., Masuda A., Mino H., Tachibana M., Inada T., Ohno K. (2024). Extremely Low-Frequency Electromagnetic Field (ELF-EMF) Increases Mitochondrial Electron Transport Chain Activities and Ameliorates Depressive Behaviors in Mice. Int. J. Mol. Sci..

[24] Kim J., Xu M., Xo R., Mates A., Wilson G.L., Pearsall A.W., Grishko V. (2010). Mitochondrial DNA Damage Is Involved in Apoptosis Caused by Pro-Inflammatory Cytokines in Human OA Chondrocytes. Osteoarthr. Cartil..

[25] Strawbridge R., Hodsoll J., Powell T.R., Hotopf M., Hatch S.L., Breen G., Cleare A.J. (2019). Inflammatory Profiles of Severe Treatment-Resistant Depression. J. Affect. Disord..

[26] Cardanho-Ramos C., Morais V.A. (2021). Mitochondrial Biogenesis in Neurons: How and Where. Int. J. Mol. Sci..

[27] Wei Y.-H., Lee H.-C. (2002). Oxidative Stress, Mitochondrial DNA Mutation, and Impairment of Antioxidant Enzymes in Aging. Exp. Biol. Med..

[28] Alexeyev M., Shokolenko I., Wilson G., LeDoux S. (2013). The Maintenance of Mitochondrial DNA Integrity—Critical Analysis and Update. Cold Spring Harb. Perspect. Biol..

[29] Feng W., Lv C., Cheng L., Song X., Li X., Xie H., Chen S., Wang X., Xue L., Zhang C. (2024). Targeting ERS-Mitophagy in Hippocampal Neurons to Explore the Improvement of Memory by Tea Polyphenols in Aged Type 2 Diabetic Rats. Free Radic. Biol. Med..

[30] Zhao Z., Xie L., Shi J., Liu T., Wang S., Huang J., Wu D., Zhang X. (2024). Neuroprotective Effect of Zishen Huoxue Decoction Treatment on Vascular Dementia by Activating PINK1/Parkin Mediated Mitophagy in the Hippocampal CA1 Region. J. Ethnopharmacol..

[31] Berlim M.T., Turecki G. (2007). What Is the Meaning of Treatment Resistant/Refractory Major Depression (TRD)? A Systematic Review of Current Randomized Trials. Eur. Neuropsychopharmacol..

[32] Rezin G.T., Amboni G., Zugno A.I., Quevedo J., Streck E.L. (2009). Mitochondrial Dysfunction and Psychiatric Disorders. Neurochem. Res..

[33] Bhattacharyya S., MahmoudianDehkordi S., Sniatynski M.J., Belenky M., Marur V.R., Rush A.J., Craighead W.E., Mayberg H.S., Dunlop B.W., Kristal B.S. (2025). Metabolomics Signatures of Serotonin Reuptake Inhibitor (Escitalopram), Serotonin Norepinephrine Reuptake Inhibitor (Duloxetine) and Cognitive-Behavioral Therapy on Key Neurotransmitter Pathways in Major Depressive Disorder. J. Affect. Disord..

[34] Deng D., Cui Y., Gan S., Xie Z., Cui S., Cao K., Wang S., Shi G., Yang L., Bai S. (2022). Sinisan Alleviates Depression-like Behaviors by Regulating Mitochondrial Function and Synaptic Plasticity in Maternal Separation Rats. Phytomedicine.

[35] Javani G., Babri S., Farajdokht F., Ghaffari-Nasab A., Mohaddes G. (2022). Mitochondrial Transplantation Improves Anxiety- and Depression-like Behaviors in Aged Stress-Exposed Rats. Mech. Ageing Dev..

[36] Mafikandi V., Seyedaghamiri F., Hosseinzadeh N., Shahabi P., Shafiee-Kandjani A.R., Babaie S., Maghsoumi-Norouzabad L., Farajdokht F., Hosseini L. (2025). Nasal Administration of Mitochondria Relieves Depressive- and Anxiety-like Behaviors in Male Mice Exposed to Restraint Stress through the Suppression ROS/NLRP3/Caspase-1/IL-1β Signaling Pathway. Naunyn-Schmiedeberg’s Arch. Pharmacol..

[37] Teng T., Shively C.A., Li X., Jiang X., Neigh G.N., Yin B., Zhang Y., Fan L., Xiang Y., Wang M. (2021). Chronic Unpredictable Mild Stress Produces Depressive-like Behavior, Hypercortisolemia, and Metabolic Dysfunction in Adolescent Cynomolgus Monkeys. Transl. Psychiatry.

[38] Ishikawa K., Yamamoto S., Hattori S., Nishimura N., Matsumoto H., Miyakawa T., Nakada K. (2021). Neuronal Degeneration and Cognitive Impairment Can Be Prevented via the Normalization of Mitochondrial Dynamics. Pharmacol. Res..

[39] Kumar P., Liu C., Suliburk J., Hsu J.W., Muthupillai R., Jahoor F., Minard C.G., Taffet G.E., Sekhar R.V. (2023). Supplementing Glycine and N-Acetylcysteine (GlyNAC) in Older Adults Improves Glutathione Deficiency, Oxidative Stress, Mitochondrial Dysfunction, Inflammation, Physical Function, and Aging Hallmarks: A Randomized Clinical Trial. J. Gerontol. Ser. A.

[40] De Jong N.P., Rudolph M.C., Jackman M.R., Sharp R.R., Jones K., Houck J., Pan Z., Reusch J.E.B., MacLean P.S., Bessesen D.H. (2022). Short-Term Adaptations in Skeletal Muscle Mitochondrial Oxidative Capacity and Metabolic Pathways to Breaking up Sedentary Behaviors in Overweight or Obese Adults. Nutrients.

[41] Fernández-de la Torre M., Fiuza-Luces C., Valenzuela P.L., Laine-Menéndez S., Arenas J., Martín M.A., Turnbull D.M., Lucia A., Morán M. (2020). Exercise Training and Neurodegeneration in Mitochondrial Disorders: Insights from the Harlequin Mouse. Front. Physiol..

[42] Tobe E.H. (2013). Mitochondrial Dysfunction, Oxidative Stress, and Major Depressive Disorder. Neuropsychiatr. Dis. Treat..

[43] Sahafi E., Peeri M., Hosseini M.-J., Azarbyjani M.A. (2018). Cardiac Oxidative Stress Following Maternal Separation Stress Was Mitigated Following Adolescent Voluntary Exercise in Adult Male Rat. Physiol. Behav..

[44] Picard M., McEwen B.S. (2018). Psychological Stress and Mitochondria: A Systematic Review. Biopsychosoc. Sci. Med..

[45] Akyuva Y., Nazıroğlu M. (2020). Resveratrol Attenuates Hypoxia-Induced Neuronal Cell Death, Inflammation and Mitochondrial Oxidative Stress by Modulation of TRPM2 Channel. Sci. Rep..

[46] Zhang H.-S., Liu C.-D., Zheng M.-C., Zhao H.-T., Liu X.-J. (2020). Propofol Alleviates Hypoxic Neuronal Injury by Inhibiting High Levels of Mitochondrial Fusion and Fission. Eur. Rev. Med. Pharmacol. Sci..

[47] Barden N. (2004). Implication of the Hypothalamic–Pituitary–Adrenal Axis in the Physiopathology of Depression. J. Psychiatry Neurosci..

[48] Druzhkova T.A., Yakovlev A.A., Rider F.K., Zinchuk M.S., Guekht A.B., Gulyaeva N.V. (2022). Elevated Serum Cortisol Levels in Patients with Focal Epilepsy, Depression, and Comorbid Epilepsy and Depression. Int. J. Mol. Sci..

[49] Thompson S.M., Kallarackal A.J., Kvarta M.D., Van Dyke A.M., LeGates T.A., Cai X. (2015). An Excitatory Synapse Hypothesis of Depression. Trends Neurosci..

[50] Ogata H., Higasa K., Kageyama Y., Tahara H., Shimamoto A., Takekita Y., Koshikawa Y., Nonen S., Kato T., Kinoshita T. (2023). Relationship between Circulating Mitochondrial DNA and microRNA in Patients with Major Depression. J. Affect. Disord..

[51] Giorgi C., Marchi S., Pinton P. (2018). The Machineries, Regulation and Cellular Functions of Mitochondrial Calcium. Nat. Rev. Mol. Cell Biol..

[52] Drevets W.C., Price J.L., Furey M.L. (2008). Brain Structural and Functional Abnormalities in Mood Disorders: Implications for Neurocircuitry Models of Depression. Brain Struct. Funct..

[53] Kondo D.G., Forrest L.N., Shi X., Sung Y.-H., Hellem T.L., Huber R.S., Renshaw P.F. (2016). Creatine Target Engagement with Brain Bioenergetics: A Dose-Ranging Phosphorus-31 Magnetic Resonance Spectroscopy Study of Adolescent Females with SSRI-Resistant Depression. Amino Acids.

[54] Wen L., Jin Y., Li L., Sun S., Cheng S., Zhang S., Zhang Y., Svenningsson P. (2014). Exercise Prevents Raphe Nucleus Mitochondrial Overactivity in a Rat Depression Model. Physiol. Behav..

[55] Huang C.-C., Wang T., Tung Y.-T., Lin W.-T. (2016). Effect of Exercise Training on Skeletal Muscle SIRT1 and PGC-1α Expression Levels in Rats of Different Age. Int. J. Med. Sci..

[56] Gureev A.P., Shaforostova E.A., Popov V.N. (2019). Regulation of Mitochondrial Biogenesis as a Way for Active Longevity: Interaction Between the Nrf2 and PGC-1α Signaling Pathways. Front. Genet..

[57] Hardie D.G. (2015). AMPK: Positive and Negative Regulation, and Its Role in Whole-Body Energy Homeostasis. Curr. Opin. Cell Biol..

[58] De Sousa R.A.L. (2024). Exercise-Produced Irisin Effects on Brain-Related Pathological Conditions. Metab. Brain Dis..

[59] Yan X., Liu J., Ye Z., Huang J., He F., Xiao W., Hu X., Luo Z. (2016). CaMKII-Mediated CREB Phosphorylation Is Involved in Ca2+-Induced BDNF mRNA Transcription and Neurite Outgrowth Promoted by Electrical Stimulation. PLoS ONE.

[60] Murphy M.P. (2008). How Mitochondria Produce Reactive Oxygen Species. Biochem. J..

[61] Turrens J.F. (2003). Mitochondrial Formation of Reactive Oxygen Species. J. Physiol..

[62] Chouchani E.T., Pell V.R., Gaude E., Aksentijević D., Sundier S.Y., Robb E.L., Logan A., Nadtochiy S.M., Ord E.N.J., Smith A.C. (2014). Ischaemic Accumulation of Succinate Controls Reperfusion Injury through Mitochondrial ROS. Nature.

[63] Kussmaul L., Hirst J. (2006). The Mechanism of Superoxide Production by NADH:Ubiquinone Oxidoreductase (Complex I) from Bovine Heart Mitochondria. Proc. Natl. Acad. Sci. USA.

[64] Pacher P., Beckman J.S., Liaudet L. (2007). Nitric Oxide and Peroxynitrite in Health and Disease. Physiol. Rev..

[65] Melo C.S., Rocha-Vieira E., Freitas D.A., Soares B.A., Rocha-Gomes A., Riul T.R., Mendonça V.A., Lacerda A.C.R., Camargos A.C.R., Carvalho L.E.D. (2019). A Single Session of High-Intensity Interval Exercise Increases Antioxidants Defenses in the Hippocampus of Wistar Rats. Physiol. Behav..

[66] Shirendeb U., Reddy A.P., Manczak M., Calkins M.J., Mao P., Tagle D.A., Hemachandra Reddy P. (2011). Abnormal Mitochondrial Dynamics, Mitochondrial Loss and Mutant Huntingtin Oligomers in Huntington’s Disease: Implications for Selective Neuronal Damage. Hum. Mol. Genet..

[67] Moore T.M., Zhou Z., Cohn W., Norheim F., Lin A.J., Kalajian N., Strumwasser A.R., Cory K., Whitney K., Ho T. (2019). The Impact of Exercise on Mitochondrial Dynamics and the Role of Drp1 in Exercise Performance and Training Adaptations in Skeletal Muscle. Mol. Metab..

[68] Innocenti G.M., Quartarone A., Ghilardi M.F., Boller F. (2022). Chapter 1—Defining Neuroplasticity. Handbook of Clinical Neurology.

[69] Ahmad R., Khan A., Rehman I.U., Lee H.J., Khan I., Kim M.O. (2022). Lupeol Treatment Attenuates Activation of Glial Cells and Oxidative-Stress-Mediated Neuropathology in Mouse Model of Traumatic Brain Injury. Int. J. Mol. Sci..

[70] Campos R.M.P., Barbosa-Silva M.C., Ribeiro-Resende V.T. (2023). A Period of Transient Synaptic Density Unbalancing in the Motor Cortex after Peripheral Nerve Injury and the Involvement of Microglial Cells. Mol. Cell. Neurosci..

[71] Durán-Carabali L.E., Odorcyk F.K., Grun L.K., Schmitz F., Ramires Junior O.V., de Oliveria M.R., Campos K.F., Hoeper E., Carvalho A.V.S., Greggio S. (2024). Maternal Environmental Enrichment Protects Neonatal Brains from Hypoxic-Ischemic Challenge by Mitigating Brain Energetic Dysfunction and Modulating Glial Cell Responses. Exp. Neurol..

[72] Si Q., Wu L., Pang D., Jiang P. (2023). Exosomes in Brain Diseases: Pathogenesis and Therapeutic Targets. MedComm.

[73] Ongnok B., Maneechote C., Chunchai T., Pantiya P., Arunsak B., Nawara W., Chattipakorn N., Chattipakorn S.C. (2022). Modulation of Mitochondrial Dynamics Rescues Cognitive Function in Rats with “doxorubicin-Induced Chemobrain” via Mitigation of Mitochondrial Dysfunction and Neuroinflammation. FEBS J..

[74] Pathak D., Shields L.Y., Mendelsohn B.A., Haddad D., Lin W., Gerencser A.A., Kim H., Brand M.D., Edwards R.H., Nakamura K. (2015). The Role of Mitochondrially Derived ATP in Synaptic Vesicle Recycling. J. Biol. Chem..

[75] Vos M., Lauwers E., Verstreken P. (2010). Synaptic Mitochondria in Synaptic Transmission and Organization of Vesicle Pools in Health and Disease. Front. Synaptic Neurosci..

[76] Torres-Platas S.G., Cruceanu C., Chen G.G., Turecki G., Mechawar N. (2014). Evidence for Increased Microglial Priming and Macrophage Recruitment in the Dorsal Anterior Cingulate White Matter of Depressed Suicides. Brain Behav. Immun..

[77] Choi G.E., Han H.J. (2021). Glucocorticoid Impairs Mitochondrial Quality Control in Neurons. Neurobiol. Dis..

[78] Clement A., Madsen M.J., Kastaniegaard K., Wiborg O., Asuni A.A., Stensballe A. (2022). Chronic Stress Induces Hippocampal Mitochondrial Damage in APPPS1 Model Mice and Wildtype Littermates. J. Alzheimer’s Dis..

[79] Wang F., Zhou T., Zhou C.X., Zhang Q.B., Wang H., Zhou Y. (2023). The Worsening of Skeletal Muscle Atrophy Induced by Immobilization at the Early Stage of Remobilization Correlates with BNIP3-Dependent Mitophagy. BMC Musculoskelet. Disord..

[80] Weger M., Alpern D., Cherix A., Ghosal S., Grosse J., Russeil J., Gruetter R., de Kloet E.R., Deplancke B., Sandi C. (2020). Mitochondrial Gene Signature in the Prefrontal Cortex for Differential Susceptibility to Chronic Stress. Sci. Rep..

[81] Csabai D., Sebők-Tornai A., Wiborg O., Czéh B. (2022). A Preliminary Quantitative Electron Microscopic Analysis Reveals Reduced Number of Mitochondria in the Infralimbic Cortex of Rats Exposed to Chronic Mild Stress. Front. Behav. Neurosci..

[82] Rial D., Lemos C., Pinheiro H., Duarte J.M., Gonçalves F.Q., Real J.I., Prediger R.D., Gonçalves N., Gomes C.A., Canas P.M. (2016). Depression as a Glial-Based Synaptic Dysfunction. Front. Cell. Neurosci..

[83] Yu L., Li Y., Lv Y., Gu B., Cai J., Liu Q.-S., Zhao L. (2024). Treadmill Exercise Facilitates Synaptic Plasticity in APP/PS1 Mice by Regulating Hippocampal AMPAR Activity. Cells.

[84] Shen W., Jin L., Zhu A., Lin Y., Pan G., Zhou S., Cheng J., Zhang J., Tu F., Liu C. (2021). Treadmill Exercise Enhances Synaptic Plasticity in the Ischemic Penumbra of MCAO Mice by Inducing the Expression of Camk2a via CYFIP1 Upregulation. Life Sci..

[85] Xie Q., Cheng J., Pan G., Wu S., Hu Q., Jiang H., Wang Y., Xiong J., Pang Q., Chen X. (2019). Treadmill Exercise Ameliorates Focal Cerebral Ischemia/Reperfusion-Induced Neurological Deficit by Promoting Dendritic Modification and Synaptic Plasticity via Upregulating Caveolin-1/VEGF Signaling Pathways. Exp. Neurol..

[86] Li R., Wang W., Guo J., Liu W., Huang X., Qiao Y., Jia S., Tian L., Zhou J., Wang G. (2023). Efficacy and Acceptability of Supervised Group Exercise for Mild to Moderate Major Depressive Disorder: A Feasibility Study. J. Affect. Disord..

[87] Swain M.K., Soman S., Tapia K., Dagda R.Y., Dagda R.K. (2023). Brain-Derived Neurotrophic Factor Protects Neurons by Stimulating Mitochondrial Function through Protein Kinase A. J. Neurochem..

[88] Molendijk M.L., Spinhoven P., Polak M., Bus B.A.A., Penninx B.W.J.H., Elzinga B.M. (2014). Serum BDNF Concentrations as Peripheral Manifestations of Depression: Evidence from a Systematic Review and Meta-Analyses on 179 Associations (N=9484). Mol. Psychiatry.

[89] Toader C., Serban M., Munteanu O., Covache-Busuioc R.-A., Enyedi M., Ciurea A.V., Tataru C.P. (2025). From Synaptic Plasticity to Neurodegeneration: BDNF as a Transformative Target in Medicine. Int. J. Mol. Sci..

[90] Hayek L.E., Khalifeh M., Zibara V., Assaad R.A., Emmanuel N., Karnib N., El-Ghandour R., Nasrallah P., Bilen M., Ibrahim P. (2019). Lactate Mediates the Effects of Exercise on Learning and Memory through SIRT1-Dependent Activation of Hippocampal Brain-Derived Neurotrophic Factor (BDNF). J. Neurosci..

[91] Sleiman S.F., Henry J., Al-Haddad R., El Hayek L., Abou Haidar E., Stringer T., Ulja D., Karuppagounder S.S., Holson E.B., Ratan R.R. (2016). Exercise Promotes the Expression of Brain Derived Neurotrophic Factor (BDNF) through the Action of the Ketone Body β-Hydroxybutyrate. eLife.

[92] Hu E., Du H., Zhu X., Wang L., Shang S., Wu X., Lu H., Lu X. (2018). Beta-Hydroxybutyrate Promotes the Expression of BDNF in Hippocampal Neurons under Adequate Glucose Supply. Neuroscience.

[93] Hu J., Cai M., Shang Q., Li Z., Feng Y., Liu B., Xue X., Lou S. (2021). Elevated Lactate by High-Intensity Interval Training Regulates the Hippocampal BDNF Expression and the Mitochondrial Quality Control System. Front. Physiol..

[94] Nasir A., Rahman M.U., Khan M., Zahid M., Shahab M., Jiao H., Zeb A., Shah S.A., Khan H. (2025). Vitamin B6 Via P-JNK/Nrf-2/NF-κB Signaling Ameliorates Cadmium Chloride-Induced Oxidative Stress Mediated Memory Deficits in Mice Hippocampus. Curr. Neuropharmacol..

[95] Didangelos T., Karlafti E., Kotzakioulafi E., Margariti E., Giannoulaki P., Batanis G., Tesfaye S., Kantartzis K. (2021). Vitamin B12 Supplementation in Diabetic Neuropathy: A 1-Year, Randomized, Double-Blind, Placebo-Controlled Trial. Nutrients.

[96] Li D., Yu S., Long Y., Shi A., Deng J., Ma Y., Wen J., Li X., Liu S., Zhang Y. (2022). Tryptophan Metabolism: Mechanism-Oriented Therapy for Neurological and Psychiatric Disorders. Front. Immunol..

[97] Castro-Portuguez R., Sutphin G.L. (2020). Kynurenine Pathway, NAD+ Synthesis, and Mitochondrial Function: Targeting Tryptophan Metabolism to Promote Longevity and Healthspan. Exp. Gerontol..

[98] Liu G., Sun W., Wang F., Jia G., Zhao H., Chen X., Tian G., Cai J., Wang J. (2023). Dietary Tryptophan Supplementation Enhances Mitochondrial Function and Reduces Pyroptosis in the Spleen and Thymus of Piglets after Lipopolysaccharide Challenge. Animal.

[99] Ciria L.F., Román-Caballero R., Vadillo M.A., Holgado D., Luque-Casado A., Perakakis P., Sanabria D. (2023). An Umbrella Review of Randomized Control Trials on the Effects of Physical Exercise on Cognition. Nat. Hum. Behav..

[100] Caponnetto P., Casu M., Amato M., Cocuzza D., Galofaro V., La Morella A., Paladino S., Pulino K., Raia N., Recupero F. (2021). The Effects of Physical Exercise on Mental Health: From Cognitive Improvements to Risk of Addiction. Int. J. Environ. Res. Public Health.

[101] Albrahim T., Alangry R., Alotaibi R., Almandil L., Alburikan S. (2023). Effects of Regular Exercise and Intermittent Fasting on Neurotransmitters, Inflammation, Oxidative Stress, and Brain-Derived Neurotrophic Factor in Cortex of Ovariectomized Rats. Nutrients.

[102] Oliveira D.M.G., Aguiar L.T., de Oliveira Limones M.V., Gomes A.G., da Silva L.C., de Morais Faria C.D.C., Scalzo P.L. (2019). Aerobic Training Efficacy in Inflammation, Neurotrophins, and Function in Chronic Stroke Persons: A Randomized Controlled Trial Protocol. J. Stroke Cerebrovasc. Dis..

[103] Vaynman S., Ying Z., Gomez-Pinilla F. (2004). Hippocampal BDNF Mediates the Efficacy of Exercise on Synaptic Plasticity and Cognition. Eur. J. Neurosci..

[104] Haghighi A.H., Bandali M.R., Askari R., Shahrabadi H., Barone R., Bei R., Farsetti P., Perrone M.A. (2025). The Effects of Different Exercise Training Protocols on Mitochondrial Dynamics in Skeletal and Cardiac Muscles of Wistar Rats. J. Orthop. Surg. Res..

[105] Kwon I., Jang Y., Lee Y. (2021). Endurance Exercise-Induced Autophagy/Mitophagy Coincides with a Reinforced Anabolic State and Increased Mitochondrial Turnover in the Cortex of Young Male Mouse Brain. J. Mol. Neurosci..

[106] Chen Z., Shen X., Tian K., Liu Y., Xiong S., Yu Q., Dai L., Shi Y., Zhang R., Zeng R. (2020). Bioavailable Testosterone Is Associated with Symptoms of Depression in Adult Men. J. Int. Med. Res..

[107] Torabi F., Ahmadi R. (2024). The Effect of Exercise Volume on Depressive-Related Behaviors and Levels of Brain-Derived Neurotrophic Factor and Serum Testosterone Levels. Int. J. Sport Stud. Health.

[108] Zhang K.J., Ramdev R.A., Tuta N.J., Spritzer M.D. (2020). Dose-Dependent Effects of Testosterone on Spatial Learning Strategies and Brain-Derived Neurotrophic Factor in Male Rats. Psychoneuroendocrinology.

[109] Stubbs B., Vancampfort D., Firth J., Schuch F.B., Hallgren M., Smith L., Gardner B., Kahl K.G., Veronese N., Solmi M. (2018). Relationship between Sedentary Behavior and Depression: A Mediation Analysis of Influential Factors across the Lifespan among 42,469 People in Low- and Middle-Income Countries. J. Affect. Disord..

[110] Hallgren M., Nguyen T.-T.-D., Owen N., Vancampfort D., Dunstan D.W., Wallin P., Andersson G., Ekblom-Bak E. (2020). Associations of Sedentary Behavior in Leisure and Occupational Contexts with Symptoms of Depression and Anxiety. Prev. Med..

[111] Endrighi R., Steptoe A., Hamer M. (2016). The Effect of Experimentally Induced Sedentariness on Mood and Psychobiological Responses to Mental Stress. Br. J. Psychiatry.

[112] Schuch F., Vancampfort D., Firth J., Rosenbaum S., Ward P., Reichert T., Bagatini N.C., Bgeginski R., Stubbs B. (2017). Physical Activity and Sedentary Behavior in People with Major Depressive Disorder: A Systematic Review and Meta-Analysis. J. Affect. Disord..

[113] Park H.-S., Kim C.-J., Kwak H.-B., No M.-H., Heo J.-W., Kim T.-W. (2018). Physical Exercise Prevents Cognitive Impairment by Enhancing Hippocampal Neuroplasticity and Mitochondrial Function in Doxorubicin-Induced Chemobrain. Neuropharmacology.

[114] Hallgren M., Nguyen T.-T.-D., Owen N., Stubbs B., Vancampfort D., Lundin A., Dunstan D., Bellocco R., Lagerros Y.T. (2020). Cross-Sectional and Prospective Relationships of Passive and Mentally Active Sedentary Behaviours and Physical Activity with Depression. Br. J. Psychiatry.

[115] Kang C., Ji L.L. (2013). Muscle Immobilization and Remobilization Downregulates PGC-1α Signaling and the Mitochondrial Biogenesis Pathway. J. Appl. Physiol..

[116] Miotto P.M., Mcglory C., Bahniwal R., Kamal M., Phillips S.M., Holloway G.P. (2019). Supplementation with Dietary ω-3 Mitigates Immobilization-Induced Reductions in Skeletal Muscle Mitochondrial Respiration in Young Women. FASEB J..

[117] Figueiredo P.A., Powers S.K., Ferreira R.M., Amado F., Appell H.J., Duarte J.A. (2009). Impact of Lifelong Sedentary Behavior on Mitochondrial Function of Mice Skeletal Muscle. J. Gerontol. Ser. A.

[118] Gungor-Orhan I., Akin S., Powers S.K., Olgaz-Bingol S., Demirel H.A. (2025). Sedentary Lifestyle Induces Oxidative Stress and Atrophy in Rat Skeletal Muscle. Exp. Physiol..

[119] Liu S., Liu X., Ke M., Wang J. (2025). Sleep Fragmentation Impairs Cognitive Function and Exacerbates Alzheimer’s Disease-Related Pathology in a Mouse Model by Disrupting Mitochondrial Biogenesis. Exp. Neurol..

[120] Fazeli Sani A., Matin Homaee H., Banaeifar A. (2020). The Effect of 4 Weeks of Aerobic Training on Spatial Learning, Memory Performance and Mitochondrial Dynamics in the Hippocampal Tissue of Old Rats. J. Ardabil Univ. Med. Sci..

[121] Demarest T.G., Varma V.R., Estrada D., Babbar M., Basu S., Mahajan U.V., Moaddel R., Croteau D.L., Thambisetty M., Mattson M.P. (2020). Biological Sex and DNA Repair Deficiency Drive Alzheimer’s Disease via Systemic Metabolic Remodeling and Brain Mitochondrial Dysfunction. Acta Neuropathol..

[122] Burbulla L.F., Song P., Mazzulli J.R., Zampese E., Wong Y.C., Jeon S., Santos D.P., Blanz J., Obermaier C.D., Strojny C. (2017). Dopamine Oxidation Mediates Mitochondrial and Lysosomal Dysfunction in Parkinson’s Disease. Science.

[123] Hyatt J.-P.K., Lu E.J., McCall G.E. (2024). Temporal Expression of Mitochondrial Life Cycle Markers during Acute and Chronic Overload of Rat Plantaris Muscles. Front. Physiol..

[124] Koo J.-H., Kang E.-B., Cho J.-Y. (2019). Resistance Exercise Improves Mitochondrial Quality Control in a Rat Model of Sporadic Inclusion Body Myositis. Gerontology.

[125] Parry H.A., Roberts M.D., Kavazis A.N. (2020). Human Skeletal Muscle Mitochondrial Adaptations Following Resistance Exercise Training. Int. J. Sports Med..

[126] Murphy J.L., Blakely E.L., Schaefer A.M., He L., Wyrick P., Haller R.G., Taylor R.W., Turnbull D.M., Taivassalo T. (2008). Resistance Training in Patients with Single, Large-Scale Deletions of Mitochondrial DNA. Brain.

[127] Franchi M., Brocca L., Sarto F., Sirago G., Candia J., Giacomello E., De Vito G., Ferrucci L., Pellegrino M.A., Bottinelli R. (2024). Resistance Exercise Following Short Term Muscle Disuse Leads to Enhanced Mitochondrial Activity and Dynamics. Physiology.

[128] Porter C., Reidy P.T., Bhattarai N., Sidossis L.S., Rasmussen B.B. (2015). Resistance Exercise Training Alters Mitochondrial Function in Human Skeletal Muscle. Med. Sci. Sports Exerc..

[129] Haun C.T., Vann C.G., Osburn S.C., Mumford P.W., Roberson P.A., Romero M.A., Fox C.D., Johnson C.A., Parry H.A., Kavazis A.N. (2019). Muscle Fiber Hypertrophy in Response to 6 Weeks of High-Volume Resistance Training in Trained Young Men Is Largely Attributed to Sarcoplasmic Hypertrophy. PLoS ONE.

[130] Chrøis K.M., Dohlmann T.L., Søgaard D., Hansen C.V., Dela F., Helge J.W., Larsen S. (2020). Mitochondrial Adaptations to High Intensity Interval Training in Older Females and Males. Eur. J. Sport Sci..

[131] Gibala M.J., Little J.P., Van Essen M., Wilkin G.P., Burgomaster K.A., Safdar A., Raha S., Tarnopolsky M.A. (2006). Short-Term Sprint Interval versus Traditional Endurance Training: Similar Initial Adaptations in Human Skeletal Muscle and Exercise Performance. J. Physiol..

[132] Jacobs R.A., Flück D., Bonne T.C., Bürgi S., Christensen P.M., Toigo M., Lundby C. (2013). Improvements in Exercise Performance with High-Intensity Interval Training Coincide with an Increase in Skeletal Muscle Mitochondrial Content and Function. J. Appl. Physiol..

[133] dos Santos J.R., Bortolanza M., Ferrari G.D., Lanfredi G.P., do Nascimento G.C., Azzolini A.E., Del Bel E., de Campos A.C., Faça V.M., Vulczak A. (2020). One-Week High-Intensity Interval Training Increases Hippocampal Plasticity and Mitochondrial Content without Changes in Redox State. Antioxidants.

[134] Little J.P., Safdar A., Wilkin G.P., Tarnopolsky M.A., Gibala M.J. (2010). A Practical Model of Low-Volume High-Intensity Interval Training Induces Mitochondrial Biogenesis in Human Skeletal Muscle: Potential Mechanisms. J. Physiol..

[135] Gibala M.J., Little J.P., MacDonald M.J., Hawley J.A. (2012). Physiological Adaptations to Low-Volume, High-Intensity Interval Training in Health and Disease. J. Physiol..

[136] Spiering B.A., Mujika I., Sharp M.A., Foulis S.A. (2021). Maintaining Physical Performance: The Minimal Dose of Exercise Needed to Preserve Endurance and Strength Over Time. J. Strength Cond. Res..

[137] Correia É.M., Monteiro D., Bento T., Rodrigues F., Cid L., Vitorino A., Figueiredo N., Teixeira D.S., Couto N. (2024). Analysis of the Effect of Different Physical Exercise Protocols on Depression in Adults: Systematic Review and Meta-Analysis of Randomized Controlled Trials. Sports Health.

[138] Wang X.-Z., Cai Z.-D., Jiang W.-T., Fang Y.-Y., Sun W., Wang X. (2022). Systematic Review and Meta-Analysis of the Effects of Exercise on Depression in Adolescents. Child Adolesc. Psychiatry Ment. Health.

[139] Cabanas-Sánchez V., Lynskey N., Ho F.K., Pell J., Celis-Morales C. (2022). Physical Activity and Risk of Depression: Does the Type and Number of Activities Matter?. Lancet.

[140] Nayor M., Shah R.V., Miller P.E., Blodgett J.B., Tanguay M., Pico A.R., Murthy V.L., Malhotra R., Houstis N.E., Deik A. (2020). Metabolic Architecture of Acute Exercise Response in Middle-Aged Adults in the Community. Circulation.

